# Turbo Gradient and Spin Echo PROPELLER-Diffusion Weighted Imaging for Orbital Tumors: A Comparative Study With Readout-Segmented Echo-Planar Imaging

**DOI:** 10.3389/fnins.2021.755327

**Published:** 2021-11-30

**Authors:** Qing Fu, Xiang-chuang Kong, Ding-Xi Liu, Kun Zhou, Yi-hao Guo, Zi-Qiao Lei, Chuan-sheng Zheng, Fan Yang

**Affiliations:** ^1^Department of Radiology, Union Hospital, Tongji Medical College, Huazhong University of Science and Technology, Wuhan, China; ^2^Hubei Province Key Laboratory of Molecular Imaging, Wuhan, China; ^3^Siemens Shenzhen Magnetic Resonance Ltd., Shenzhen, China; ^4^MR Collaboration, Siemens Healthcare Ltd., Guangzhou, China

**Keywords:** diffusion weighted imaging, apparent diffusion coefficient, orbit tumor, turbo gradient and spin echo PROPELLER diffusion-weighted imaging (TGSE-PROPELLER-DWI), readout-segmented echo-planar imaging (rs-EPI)

## Abstract

**Purpose:** To qualitatively and quantitatively compare the image quality and diagnostic performance of turbo gradient and spin echo PROPELLER diffusion-weighted imaging (TGSE-PROPELLER-DWI) vs. readout-segmented echo-planar imaging (rs-EPI) in the evaluation of orbital tumors.

**Materials and Methods:** A total of 43 patients with suspected orbital tumors were enrolled to perform the two DWIs with comparable spatial resolution on 3T. The overall image qualities, geometric distortions, susceptibility artifacts, and lesion conspicuities were scored by using a four-point scale (1, poor; 4, excellent). Quantitative measurements, including contrast-to-noise ratios (CNRs), apparent diffusion coefficients (ADCs), geometric distortion rates (GDRs), and lesion sizes, were calculated and compared. The two ADCs for differentiating malignant from benign orbital tumors were evaluated. Wilcoxon signed-rank test, Kappa statistic, and receiver operating characteristics (ROC) curves were used.

**Results:** TGSE-PROPELLER-DWI performed superior in all subjective scores and quantitative GDR evaluation than rs-EPI (*p* < 0.001), and excellent interobserver agreement was obtained for Kappa value ranging from 0.876 to 1.000. ADC_*lesion*_ of TGSE-PROPELLER-DWI was significantly higher than those of rs-EPI (*p* < 0.001). Mean ADC of malignant tumors was significantly lower than that of benign tumors both in two DWIs. However, the AUC for differentiating malignant and benign tumors showed no significant difference in the two DWIs (0.860 vs. 0.854, *p* = 0.7448). Sensitivity and specificity could achieve 92.86% and 72.73% for TGSE-PROPELLER-DWI with a cutoff value of 1.23 × 10^–3^ mm^2^/s, and 85.71% and 81.82% for rs-EPI with a cutoff value of 0.99 × 10^–3^ mm^2^/s.

**Conclusion:** Compared with rs-EPI, TGSE-PROPELLER-DWI showed minimized geometric distortion and susceptibility artifacts significantly improved the image quality for orbital tumors and achieved comparable diagnostic performance in differentiating malignant and benign orbital tumors.

## Introduction

Diffusion weighted imaging (DWI) is an essential MR sequence for diagnosing a broad spectrum of orbital diseases, enabling characteristics on morphological features of tissues with measurements of apparent diffusion coefficient (ADC). ADC is an additional noninvasive parameter for differentiation of malignant orbital tumors from benign lesions (Sepahdari et al., 2010; Razek et al., 2011; Intven et al., 2014).

The common single-shot echo-planar DWI (SS-EPI-DWI) collects k-space data with an echo-planar imaging (EPI) trajectory and has advantages of fast acquisition. However, it is very vulnerable to susceptibility artifacts, especially for the structures with air-bone-tissue interfaces because of relative long readout time and low bandwidth in phase-encoding direction (Turner et al., 1990; Li et al., 2015). Thus, SS-EPI-DWI usually results in unsatisfactory image quality with severe geometric distortions, signal pile-up or ghosting artifacts, and even misdiagnosis of anatomical structures/abnormalities, and lesions with small and/or irregular contour are difficult to be evaluated (White et al., 2006; Heidemann et al., 2010; Fu et al., 2021). Readout-segmented echo-planar imaging (rs-EPI) is an advanced DWI technique, in which the k-space is divided into several segments along the readout direction, and echo spacing and echo time were reduced (Porter and Heidemann, 2009). The previous studies (Bogner et al., 2012; Koyasu et al., 2014; Friedli et al., 2015; Xia et al., 2016; Zhao et al., 2016; Xu et al., 2017) demonstrated that rs-EPI showed reduced susceptibility-induced artifacts and T2^∗^ blurring compared with SS-EPI-DWI. However, it is unavoidable to suffer from the image distortions and susceptibility-induced artifacts (Bogner et al., 2012; Koyasu et al., 2014; Zhao et al., 2016; Xu et al., 2017; Sheng et al., 2020), challenging to distinguish small lesions from artifacts (Sheng et al., 2020).

Turbo gradient and spin echo PROPELLER diffusion-weighted imaging (TGSE-PROPELLER-DWI) is proposed recently, and its basic imaging principles have been introduced by Li et al. (2011); Zhou et al. (2018), and Hu et al. (2019). The fast turbo-gradient and spin-echo (TGSE) readout method in this technique helps reduce image distortions and magnetic susceptibility-induced artifacts; the phase-insensitive preparation module between diffusion preparation and the data acquisition was used to eliminate non-CPMG signal component; the gradient-spin echo is placed into each independent blade to minimize dephasing artifacts; the data is collected by rotating the blades, and the image is reconstructed with phase-error correction, achieving better robustness to motion artifacts. However, the signal-to-noise ratio (SNR) efficiency of TGSE-PROPELLER-DWI maybe lower than that of rs-EPI. It might be possible to reduce the acquisition time of TGSE-PROPELLER-DWI by using coils with higher channels, higher magnet field strength, and/or higher SNR (the parameters need to be optimized accordingly).

In clinical practice, TGSE-PROPELLER-DWI has been applied in the pediatric brain (Hu et al., 2019) and has also been previously compared with rs-EPI in clinical applications of middle ear (Sheng et al., 2020) and optic nerve (Yuan et al., 2020). Hu et al. (2019) suggested that TGSE-PROPELLER-DWI should be the first choice in patients where significant magnetic susceptibility artifacts are foreseen in some specific cases, for example, patients with orthodontia, postsurgical resection cavity, or shunts which are more susceptible to yield susceptibility artifacts. Sheng et al. (2020) and Yuan et al. (2020) found that TGSE-PROPELLER-DWI significantly improved image qualities by reducing geometric distortions, susceptibility artifacts, and less blurring compared with rs-EPI images in diagnosis of cholesteatoma and optic neuritis, but with a lower signal-to-noise ratio (SNR) than that of rs-EPI. However, the performance of TGSE-PROPELLER-DWI in depicting orbital tumors is still unknown. Whether it could decrease the geometric distortions and susceptibility artifacts or not is important to depict some small lesions, the extent of invasion and the diffusion characterization of orbital tumors accurately, and is also essential for the clinical treatment planning or even prognosis of the orbital tumors.

The purpose of the current study was to compare the clinical utility of TGSE-PROPELLER-DWI for depicting orbital tumors compared with rs-EPI. Specifically, image qualities of TGSE-PROPELLER-DWI and rs-EPI were compared subjectively and objectively, and their capacity for differentiating malignant from benign tumors was also evaluated.

## Materials and Methods

### Study Population

This current prospective study was approved by the local medical ethics committee, and written informed consent was obtained prior to the MRI examinations. One of the patients was only 4 years old, and written informed consent was obtained from his legal guardians. Between September 1, 2020 and March 15, 2021, 43 patients (22 males and 21 females; mean age, 54.2 years; age range, 4–75 years) with suspected orbital tumors were enrolled in this study. Of those patients, 23 patients (10 males and 13 females; mean age, 49.3 years; age range, 4–69 years) with malignant tumors and 14 patients (10 males and 4 females; mean age, 60.6 years; age range, 49–71 years) with benign tumors were identified by pathological results 1–5 days after MR scanning. The remaining six patients were not found to have any orbital abnormalities.

Twenty-three benign orbital lesions comprised inflammatory pseudotumor (*n* = 5), cavernous malformation (*n* = 9), benign cyst (*n* = 3), dermoid cyst (*n* = 1), schwannoma (*n* = 2), meningioma (*n* = 2), and mucous cyst (*n* = 1). Fourteen malignant orbital lesions comprised lymphoma (*n* = 10), adenoid cystic carcinoma (*n* = 1), melanoma (*n* = 1), malignant solitary fibrous tumor (*n* = 1), and inflammatory myofibroblastic tumor (*n* = 1).

### Magnetic Resonance Imaging Protocol

All MRI examinations were performed on a 3T MR scanner (MAGNETOM Skyra, Siemens Healthcare, Germany) equipped with a 45 mT/m achievable gradient strength and 200 T/m/s maximum slew rate using a dedicated 20-channel head-neck coil. Conventional MRI was required using the following sequences: coronal, axial and sagittal turbo spin-echo (TSE) T2-weighted imaging (T2WI) with fat suppression [repetition time/echo time (TR/TE): 5,220/37 ms, FOV: 200 mm × 200 mm, bandwidth: 220 Hz/pixel, slice thickness: 3 mm, flip angle = 160°, resolution 320 × 320, acquired voxel size = 0.63 mm × 0.63 mm × 3.0 mm]; axial and coronal TSE T1-weighted imaging (T1WI) without fat suppression (TR/TE: 600/6.4 ms, FOV: 180 mm × 180 mm, bandwidth: 391 Hz/pixel, slice thickness: 3 mm, flip angle = 150°, acquired voxel size = 0.35 mm × 0.35 mm × 3.0 mm); axial T1WI scanned by volume interpolated body examination (VIBE) with fat suppression before and after administration of contrast material (TR/TE: 18/3.69 ms, FOV: 180 mm × 180 mm, bandwidth: 180 Hz/pixel, slice thickness: 1 mm, flip angle = 9°, acquired voxel size = 0.63 mm × 0.63 mm × 1 mm).

Before contrast injection, the prototype TGSE-PROPELLER-DWI sequence and product rs-EPI were scanned with comparable spatial resolutions and similar acquisition time for all the enrolled patients. The detailed TGSE-PROPELLER-DWI parameters were as follows: TR/TE: 4,400/59 ms, FOV: 230 mm × 230 mm, bandwidth: 650 Hz/pixel, voxel size: 1.2 mm × 1.2 mm × 2.5 mm, number of slices: 16, matrix size: 192 × 192, PROPELLER coverage: 214.3%, PROPELLER number: 30, EPI factor: 5, turbo factor: 11, echo spacing: 13.9 ms, diffusion encoding mode: 4-scan-trace, *b*-values: 0 and 1,000 s/mm^2^, averages: 1 for *b* = 0 s/mm^2^ and 3 for *b* = 1,000 s/mm^2^, turbo factor: 11, EPI factor: 5, and acquisition time: 5 min 51 s. The detailed parameters for rs-EPI were as follows: TR/TE: 6,670/68 ms, FOV: 200 mm × 200 mm, bandwidth: 780 Hz/pixel, voxel size: 1.1 mm × 1.1 mm × 2.5 mm, number of slices: 16, matrix size: 178 × 178, echo spacing: 0.4 ms, readout segments: 5, readout partial Fourier (Frost et al., 2012) was used: 3 readout segments acquired of 5 total (readout partial Fourier 5/8), generalized autocalibrating partially parallel acquisition (GRAPPA) was implemented to save time and reduce the effective echo-spacing, GRAPPA R:2, EPI factor: 89, diffusion encoding mode: 4-scan-trace, *b*-values: 0 and 1,000 s/mm^2^, averages: 1 for *b* = 0 s/mm^2^ and 3 for *b* = 1,000 s/mm^2^, and acquisition time: 5 min 35 s. The phase-encoding directions for the two DWI methods were both set to be left-right.

### Subjective Evaluations

Two experienced radiologists (with 15 and 25 years of experience working in neuroradiology) independently scored geometric distortions, susceptibility artifacts, lesion conspicuities, and the overall image qualities using a 4-point scale, as listed in Table 1. All the conventional T1WI and T2WI images were shown to the two radiologists, but images of TGSE-PROPELLER-DWI and rs-EPI of each patient were shown blindly. The order of patients and sequences (TGSE-PROPELLER-DWI or rs-EPI) was arranged in a random manner.

**TABLE 1:** Criteria for qualitative image quality comparisons between two DWIs.

**Geometric distortion**
1. Severe distortion
2. Moderate distortion
3. Mild distortion
4. No distortion
**Susceptibility artifacts**
1. Severe artifacts
2. Major artifacts
3. Only minor artifacts
4. No artifacts
**Lesion conspicuity**
1. Unable to evaluate
2. Acceptable for visualization
3. Obvious visibility
4. Excellent for visualization
**Overall image quality**
1. Poor, insufficient for diagnosis
2. Fair, adequate for diagnosis
3. Good for diagnosis
4. Excellent for diagnosis

### Objective Evaluations

Quantitative measurements, including contrast-to-noise ratios (CNRs), and apparent diffusion coefficients (ADCs), geometric distortion rates (GDRs), and lesion sizes of the two DWI sequences were calculated and compared.

The value of CNR was calculated by the following formula (Bogner et al., 2012): $\text{CNR}=\frac{{\text{SI}}_{\text{lesion}}-{\text{SI}}_{\text{WM}}}{\sqrt{{\left({\mathrm{SD}}_{\text{lesion}}\right)}^{2}+{\left({\text{SD}}_{\text{WM}}\right)}^{2}}}$, SI_*lesion*_ and SI_*WM*_ represent the mean signal intensities of lesions and white matter.

In here, SD_lesion_ and SD_WM_ represent the standard deviation of lesions and white matter.

Apparent diffusion coefficients of orbital tumors were measured in ADC maps of both DWIs. The matching circular region-of-interest (ROI) on orbital tumors in the rs-EPI ADC map was copied to the corresponding region in the TGSE-PROPELLER-DWI ADC map, and the placement of ROIs would be moved slightly in order to achieve the matching region between the two DWI methods. Then, the mean ADC values were generated automatically and recorded. All measurements were performed, avoiding signals from adjacent air, bone, susceptibility artifacts, and cystic or necrotic components of tumors.

**FIGURE 1:**
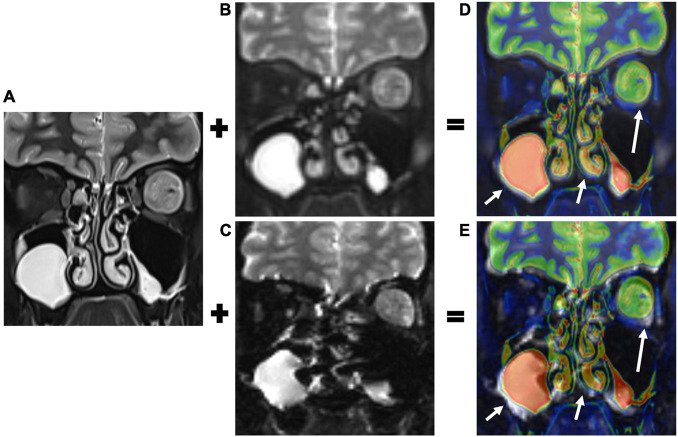
T2-weighted imaging (T2WI, **A**), b0 images of TGSE-PROPELLER-DWI **(B)**, and rs-EPI **(C)** were fused. Color- and gray-coated images were derived from the T2WI and the two DWI b0 images **(D,E)**, respectively. The T2WI and TGSE-PROPELLER-DWI b0 images matched well, showing the left orbital tumor (long arrows in **D**) and ethmoid and maxillary sinuses (short arrows in **D**). The T2WI and rs-EPI b0 images were mismatched due to geometric distortions in the corresponding structures (arrows in **E**).

Image fusions between the T2-weighted imaging (T2WI) and the two DWI sequences (*b* = 0 s/mm^2^) were used as references for grading the extent of geometric distortions which was similar to strategies used by previous studies (Koyasu et al., 2014; Zhao et al., 2016; Figure 1). The value of GDR (Yokohama et al., 2019) was calculated as follows:


GDR=(|L-DWIL|T2)/L×T2100%

Where L_*T2*_ and L_*DWI*_ represent the maximum length of the vitreous body in T2WI and DWI, respectively. For lesion size, the maximum transversal and longitudinal length of tumors were measured in coronal T2WI, rs-EPI, and TGSE-PROPELLER-DWI images. The phase-encoding directions for the two DWI methods were both set to be left-right, so geometric distortion was mainly shown in this direction (left-right anatomically) especially in rs-EPI.

### Statistical Analysis

Continuous variables were presented as the mean ± standard deviation (SD). If the subjective and objective parameters of the two DWIs conformed to normal distributions, paired *t*-test was used; otherwise, the Wilcoxon signed-rank test was used. Pearson correlation coefficients (*r*) were used to evaluate the correlations of lesion size on the two DWIs with that of T2WI. Inter-observer agreement between the two radiologists for the scores was analyzed using Kappa statistic (<0.40, poor; 0.40–0.59, moderate; 0.60–0.75, good; >0.75, excellent). Receiver operating characteristics (ROC) curves were used to evaluate the diagnostic performance of ADC values for differentiating orbital benign and malignant tumors, and the method by DeLong et al. (1988) was used to compare the area under curves (AUCs) of the two DWIs with 95% confidence intervals (CIs). Statistical analyses were performed with SPSS software (IBM SPSS 22, IBM Corp., Armonk, NY, United States) and MedCalc software (version 18.11.3; MedCalc Software, Ltd). A *p*-value <0.05 was considered statistically significant.

## Results

All the orbital lesions were detected successfully by the two DWIs. Mean lesion size was 1.93 cm × 1.86 cm in T2WI, 1.88 cm × 1.89 cm in TGSE-PROPELLER-DWI, and 1.91 cm × 1.91 cm in rs-EPI.

### Subjective Scores and Inter-Observer Agreement Evaluations

**FIGURE 2:**
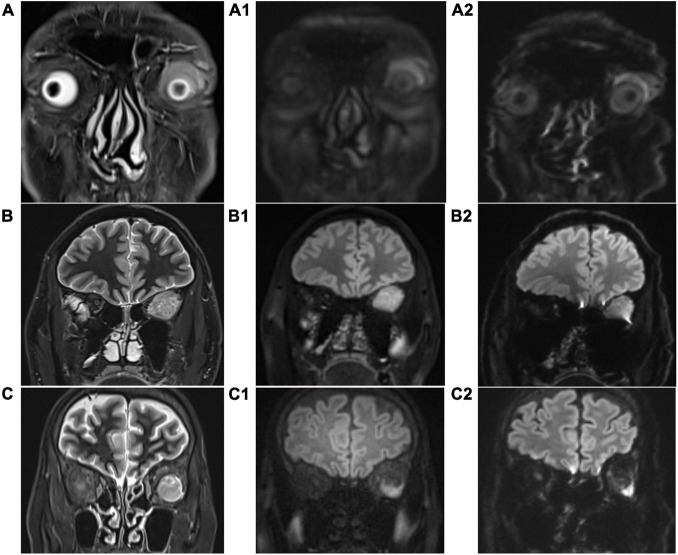
Coronal T2-weighted imaging (T2WI) images **(A–C)**, coronal TGSE-PROPELLER-DWI b1000 images **(A1–C1)**, and coronal rs-EPI b1000 images **(A2–C2)**. **(A,A1,A2)**: patient 1, male, 62 years old, left orbital lymphoma, scores of geometric distortions, susceptibility artifacts, lesion conspicuity, and overall image quality of TGSE-BLADE-DWI were 4, 4, 4, and 4, respectively, while those of rs-EPI were 3, 2, 3, and 3, respectively. **(B,B1,B2)**: patient 2, male, 50 years old, left orbital fibroma, scores of geometric distortions, susceptibility artifacts, lesion conspicuity, and overall image quality of TGSE-BLADE-DWI were 4, 4, 4, and 4, respectively, while those of rs-EPI were 3, 3, 4, and 4, respectively. **(C,C1,C2)**: patient 3, female, 61 years old, left orbital hemangioma, scores of geometric distortions, susceptibility artifacts, lesion conspicuity, and overall image quality of TGSE-BLADE-DWI were 4, 4, 4, and 4, respectively, while those of rs-EPI were 2, 3, 2, and 2, respectively.

As shown in Table 2 and Figure 2, scores in TGSE-PROPELLER-DWI were significantly higher than those in rs-EPI for geometric distortions (3.9 ± 0.21, 2.14 ± 0.71, *z* = −8.22, *p* < 0.001), susceptibility artifacts (3.91 ± 0.36, 2.14 ± 0.60, *z* = −8.32, *p* < 0.001), lesion conspicuity (3.80 ± 0.52, 3.09 ± 0.88, *z* = −5.50, *p* < 0.001), and overall image quality (3.90 ± 0.31, 2.67 ± 0.52, *z* = −8.20, *p* < 0.001). No statistical difference in the interobserver variability for the qualitative scores was observed between the two radiologists (*p*-values >0.150). In addition, the Kappa value was 0.876–1.000, thus indicating excellent interobserver agreement.

**TABLE 2:** Qualitative scores for assessing image quality of the two DWIs.

**Quantitative parameters**	**Reader 1**				**Reader 2**				**Inter-reader agreement (Kappa value)**	
	**TGSE-PROPELLER-DWI**	**rs-EPI**	***z***	***P***	**TGSE-PROPELLER-DWI**	**rs-EPI**	***z***	***P***	**TGSE-PROPELLER-DWI**	**rs-EPI**
Geometric distortion	3.95 ± 0.21	2.12 ± 0.73	−5.82	<0.001	3.95 ± 0.21	2.16 ± 0.69	−5.84	<0.001	1.000	0.925
Susceptibility artifacts	3.91 ± 0.37	2.12 ± 0.63	−5.89	<0.001	3.91 ± 0.37	2.16 ± 0.57	−5.90	<0.001	1.000	0.912
Lesion conspicuity	3.81 ± 0.52	3.11 ± 0.88	−3.98	<0.001	3.78 ± 0.53	3.08 ± 0.89	−3.84	<0.001	0.898	0.960
Overall image quality	3.91 ± 0.29	2.67 ± 0.52	−5.85	<0.001	3.89 ± 0.32	2.67 ± 0.52	−5.77	<0.001	0.876	1.000

### Objective Analyses

**FIGURE 3:**
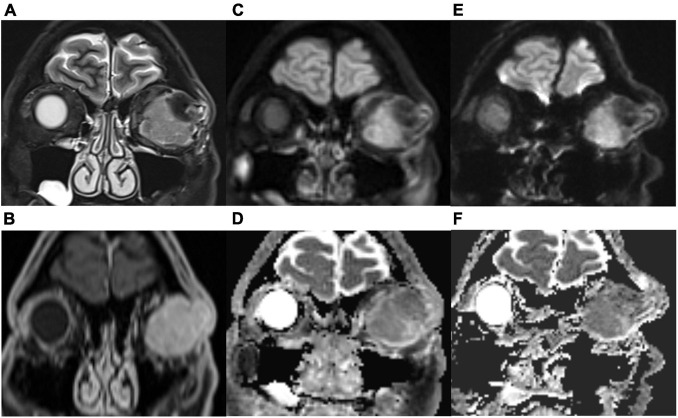
A 50-year-old male with left solitary fibrous tumor. Compared with the T2-weighted imaging (T2WI, **A**) images and contrast-enhanced T1WI **(B)**, this lesion could be visualized clearly with TGSE-PROPELLER-DWI b1000 **(C)** and ADC map **(D)**; no geometric distortions were seen. Slight distortions were seen with rs-EPI b1000 **(E)** and ADC map **(F)**. More geometric distortions were observed in the frontal lobe and ethmoid sinus with rs-EPI.

As shown in Table 3, TGSE-PROPELLER-DWI showed significantly reduced geometric distortions compared with rs-EPI for quantitative GDR evaluation (*p* < 0.001) (Figure 3, arrows); there was no significant difference in orbital tumor CNRs and white matter ADCs between the two DWI sequences (*p* = 0.085, *p* = 0.110, respectively); lesion ADC values of TGSE-PROPELLER-DWI were significantly higher than those of rs-EPI (*p* < 0.001). Figure 3 shows a left orbital tumor (identified as solitary fibrous tumor by pathological result) which could be visualized clearly using the two DWIs. TGSE-PROPELLER-DWI showed minimized distortions and less susceptibility artifacts compared to rs-EPI. GDR values on TGSE-PROPELLER-DWI and rs-EPI were 0.036 and 0.171, respectively. ADC values of solid components on TGSE-PROPELLER-DWI and rs-EPI were 1.24 × 10^–3^ mm^2^/s and 1.11 × 10^–3^ mm^2^/s.

**TABLE 3:** Quantitative parameters between TGSE-PROPELLER-DWI and rs-EPI.

**Quantitative parameters**	**TGSE-PROPELLER-DWI**	**rs-EPI**	***Z-*value**	***P-*value**
**ADC (×10^–3^ mm^2^/s)**				
White matter	0.78 ± 0.05	0.77 ± 0.05	–1.600	0.110
Lesion	1.23 ± 0.54	1.15 ± 0.56	–3.708	< 0.001
CNR (*b* = 1,000 s/mm^2^)	5.36 ± 4.82	4.13 ± 4.04	–1.720	0.085
GDR (%)	3.61 ± 3.58	17.14 ± 15.31	–5.580	< 0.001
**Lesion size (mm)**				
Maximum transversal length	18.88 ± 9.21	19.13 ± 9.80	–0.770	0.441
Maximum longitudinal length	18.91 ± 10.07	19.08 ± 9.82	–1.001	0.317

Correlation of TGSE-PROPELLER-DWI with conventional T2WI on the maximum transversal length and maximum longitudinal length (*r* = 0.986, *r* = 0.969, respectively) were both better than those of rs-EPI (*r* = 0.941, *r* = 0.960, respectively; Table 4). There was no significant difference in tumor size between TGSE-PROPELLER-DWI and rs-EPI (*p* > 0.30).

**TABLE 4:** Correlations of TGSE-PROPELLER-DWI and rs-EPI with conventional T2WI for evaluation of orbital tumor size.

**Type**	**Maximum transversal length**		**Maximum longitudinal length**	
	* *r*	***p***	* *r*	***p***
TGSE-PROPELLER-DWI	0.986	<0.001	0.969	<0.001
rs-EPI	0.941	<0.001	0.960	<0.001

*r, Pearson correlation coefficient.

### Apparent Diffusion Coefficient Analyses for Differentiating Malignant From Benign Orbital Tumors

**FIGURE 4:**
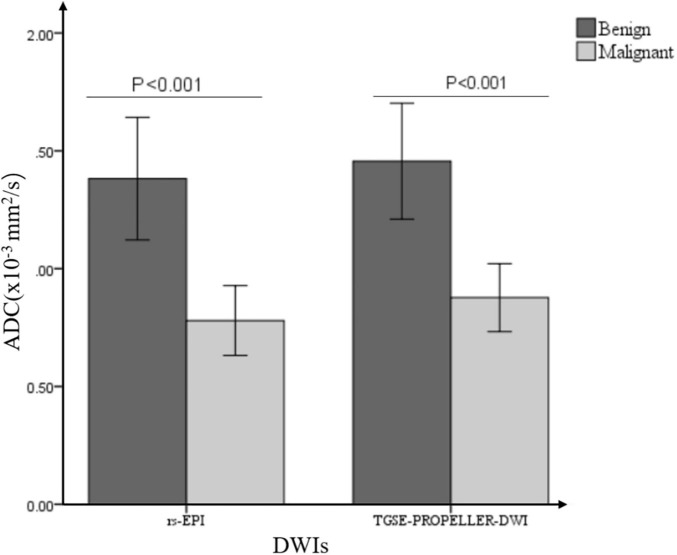
Bar graph demonstrated the comparisons of ADC values between malignant and benign orbital tumors in two DWIs.

**FIGURE 5:**
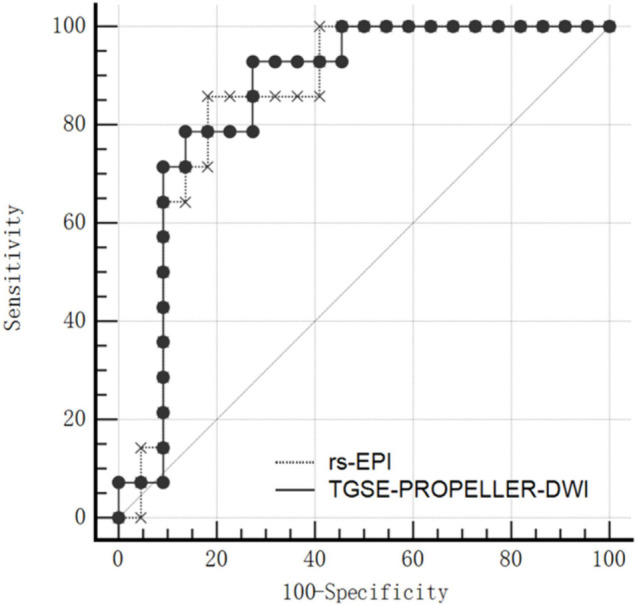
ROC curve of the two DWI ADC values to differentiate malignant and benign orbital tumors.

Mean ADC value of malignant tumors was significantly lower than that of benign tumors both in TGSE-PROPELLER-DWI (0.88 ± 0.25 × 10^–3^ mm^2^/s, 1.46 ± 0.55 × 10^–3^ mm^2^/s, *z* = −2.86, *p* = 0.004) and in rs-EPI (0.78 ± 0.26 × 10^–3^ mm^2^/s, 1.38 ± 0.59 × 10^–3^ mm^2^/s, *z* = −2.79, *p* = 0.005) (Figure 4). Even though AUC value of TGSE-PROPELLER-DWI was slightly larger than that of rs-EPI (0.860 vs. 0.854, Figure 5 and Table 5), there was no significant difference in the AUCs for both DWIs (*z* = 0.326, *p* = 0.7448). Sensitivity and specificity could achieve 92.86% and 72.73% for TGSE-PROPELLER-DWI (cutoff value: 1.23 × 10^–3^ mm^2^/s), and 85.71% and 81.82% for rs-EPI (cutoff value: 0.99 × 10^–3^ mm^2^/s) (Table 5).

**TABLE 5:** Receiver operating characteristic (ROC) curve analyses of ADC values for distinguishing orbital malignancy from benign tumors.

**DWIs**	**AUC**	**95% CI**	***z***	***P*-value**	**Youden index**	**Cutoff value (×10^–3^ mm^2^/s)**	**Sensitivity (95% CI)**	**Specificity (95% CI)**
TGSE-PROPELLER-DWI	0.860	0.704–0.953	5.504	<0.0001	0.6558	1.23	92.86% (66.1–99.8)	72.73% (49.8–89.3)
rs-EPI	0.854	0.696–0.949	5.326	<0.0001	0.6753	0.99	85.71% (57.2–98.2)	81.82% (59.7–94.8)

*AUC, area under curve; 95% CI, 95% confidence intervals.*

## Discussion

This study demonstrated the feasibility and diagnostic performance of TGSE-PROPELLER-DWI for orbital tumors in clinical use. The data supported TGSE-PROPELLER-DWI to be superior than rs-EPI with regard to improved image quality and comparable capacity for differentiating malignant and benign orbital tumors.

In orbital DWI, TGSE-PROPELLER-DWI presented with minimized geometric distortions and susceptibility artifacts when compared with rs-EPI. According to various comparison studies between rs-EPI and SS-EPI-DWI, rs-EPI produced less image distortions, image blurring, and susceptibility artifacts in applications of orbital masses, sinonasal lesions, rectal cancer, head and neck, breast cancer, renal DWI by using semiquantitative scales or quantitative scores (Bogner et al., 2012; Koyasu et al., 2014; Friedli et al., 2015; Xia et al., 2016; Zhao et al., 2016; Xu et al., 2017), as well as increased sharpness of rs-EPI (Friedli et al., 2015; Zhao et al., 2016; Xu et al., 2017). However, partial volume effect, T2^∗^ blurring effect, and geometric distortions could not be completely removed in rs-EPI (Bogner et al., 2012; Koyasu et al., 2014; Zhao et al., 2016; Xu et al., 2017; Sheng et al., 2020), which may lead to the failure to detect some small lesions (<2.5 mm) such as cholesteatomas (Sheng et al., 2020), because it was difficult to distinguish the small-sized lesions with the interference of susceptibility artifacts, especially in the air-tissue and bone-tissue interfaces. While TGSE-PROPELLER-DWI was immune to those artifacts in current study, subjective and objective assessments showed the minimized distortions and susceptibility artifacts of TGSE-PROPELLER-DWI for orbital DWI than rs-EPI, leading to the improved ability for depicting anatomical structures and lesions than rs-EPI. This result was consistent with a recent study, which focused on TGSE-PROPELLER-DWI for middle ear cholesteatoma with rs-EPI (Sheng et al., 2020) and another study which focused on TGSE-PROPELLER-DWI for the pediatric brain with SS-EPI-DWI (Hu et al., 2019). Different from the previous studies (Hu et al., 2019; Sheng et al., 2020), we also performed quantitative evaluations for geometric distortions. GDR of TGSE-PROPELLER-DWI was significantly lower than that of rs-EPI (0.036 vs. 0.171, *p* < 0.001) in the current study, which was in agreement with the subjective scores, supporting the superior performance of TGSE-PROPELLER-DWI with reduced distortions than rs-EPI.

Even though DWI could observe and quantify biological structures based on diffusion properties of water molecules and improve lesion conspicuity (Prezzi et al., 2018), it has limited role for MR-guided planning in clinical practice mainly due to disadvantages in geometric integrity (Liney and Moerland, 2014). Decreased geometric distortions and susceptibility artifacts of DWI are essential for radiotherapy (RT) in matching or image fusions with other anatomical images for external beam planning, because high degree of accuracy is needed in RT applications (Bergen et al., 2020). Minimized geometric distortion is of critical importance to avoid excessive radiation dose to healthy tissues (Bergen et al., 2020). According to several published literatures (Chen et al., 2006; McPartlin et al., 2016; Studenski et al., 2016; Bergen et al., 2020), approximately 3 mm distortion would increase the doses in RT, and it would lead to significant dose errors if more than 1 cm and even affects the treatment outcomes. Results of the current study showed the superiority of TGSE-PROPELLER-DWI with minimized distortions and susceptibility artifacts, indicating the potential to be implemented in RT management. It would be meaningful to validate its performance and accuracy in the further research.

In orbital DWI, TGSE-PROPELLER-DWI was comparable in depicting tumor size and showed better lesion conspicuity when compared with rs-EPI. Even though the correlation of the lesion size in TGSE-PROPELLER-DWI with conventional T2WI was better than in rs-EPI-DWI, there was no significant difference in lesion sizes between the two DWIs, providing additional evidence of the new TGSE-PROPELLER-DWI for clinical use with the equivalent performance in depicting lesion sizes. With the help of the decreased artifacts and distortions mentioned above, lesions with better conspicuity and comparable CNR were observed in TGSE-PROPELLER-DWI, making a greatly improved image quality of TGSE-PROPELLER-DWI to rs-EPI in orbital imaging.

In orbital DWI, TGSE-PROPELLER-DWI ADCs could achieve comparable diagnostic performance for differentiation malignant from benign orbital tumors than rs-EPI ADCs. ADC value has been widely accepted as an effective biomarker for differential diagnosis of malignant and benign tumors in various body parts (Tsuruda et al., 1990; Yamasaki et al., 2005; Maeda and Maier, 2008; Sepahdari et al., 2010). Malignant tumors with restricted diffusion tend to have a lower ADC value, which may be related to their larger nuclei, higher cellularity, and decreased extracellular space, and benign tumors tend to display higher ADCs than malignant tumors (Tsuruda et al., 1990; Guo et al., 2002; EI Khouli et al., 2010). Our results demonstrated that the mean ADC value of malignant tumors was 0.88 ± 0.25 × 10^–3^ mm^2^/s in TGSE-PROPELLER-DWI and 0.78 ± 0.26 × 10^–3^ mm^2^/s in rs-EPI, and benign tumor was 1.46 ± 0.55 × 10^–3^ mm^2^/s in TGSE-PROPELLER-DWI and 1.38 ± 0.59 × 10^–3^ mm^2^/s in rs-EPI. This finding was in good agreement with published papers. Mean ADC of the two DWIs for malignant and benign orbital tumors was 0.84 ± 0.34 × 10^–3^ mm^2^/s and 1.57 ± 0.33 × 10^–3^ mm^2^/s, respectively, in Razek et al.’s (2011) report and 0.77 ± 0.38 × 10^–3^ mm^2^/s and 1.23 ± 0.42 × 10^–3^ mm^2^/s, respectively, in Fatima et al.’s (2014) report.

Several previous orbital DWI studies have reported different threshold values with different sensitivities and specificities. Razek et al. (2011) reported a cutoff value of 1.15 × 10^–3^ mm^2^/s, with 95% sensitivity and 91% specificity for differentiating malignancy from benign lesions. Hemat (2017) reported cutoff value of 0.93 × 10^–3^ mm^2^/s with 80% sensitivity, 83.3% specificity and 82% accuracy. Sepahdari et al. (2010) reported a threshold value of 1.0 × 10^–3^ mm^2^/s with 63% sensitivity and 86% specificity. Bogner et al. (2012) reported an ADC threshold of 1.25 × 10^–3^ mm^3^/s with 90% accuracy for discriminating malignant and benign lesions. While in the current study, when TGSE-PROPELLER-DWI ADC value of 1.23 × 10^–3^ mm^2^/s was used as a cutoff value for differentiation of malignant from benign orbital tumors, sensitivity and specificity could achieve 92.86% and 72.73%, respectively, and rs-EPI ADC cutoff value was 0.99 × 10^–3^ mm^2^/s with sensitivity 85.71% and specificity 81.82%.

Moreover, in the current work, the AUC value of TGSE-PROPELLER-DWI (AUC = 0.860) was similar to that of rs-EPI (AUC = 0.854) with no significant statistical difference (*p* > 0.700), indicating a comparable diagnostic performance of the two DWIs for malignant and benign tumors differentiation. This data validated TGSE-PROPELLER-DWI to be a potential tool for characterizing orbital tumors as well as valuable differentiation of malignant from benign orbital tumors.

Our study had several limitations. First, the level of geometric distortion and susceptibility artifacts in rs-EPI is directly controlled by the echo-spacing which was set to 0.40 ms in this study where the scan time and number of averages were matched. Depending on other protocol details, e.g., decrease resolution or bandwidth, increase number of readout segment, the echo-spacing can be decreased which would reduce distortion and susceptibility artifacts. Second, we have set averages 1 for *b* = 0 s/mm^2^ and 3 for *b* = 1,000 s/mm^2^ in two DWIs by taking into account the specific characteristics of orbit for better image qualities, and acquisition times of TGSE-PROPELLER-DWI and rs-EPI were both 5–6 min. The longer examination time may yield unnecessary patient motion, resulting in motion artifacts and deterioration in image quality. Theoretically, TGSE-PROPELLER-DWI has the potential to reduce the motion artifacts to some extent due to the PROPELLER technique, but this was not investigated in current study. Further optimization of the sequence will be undertaken to decrease the scanning time for faster scanning procedure and patients’ comfort, by integrating simultaneous multislice (SMS) or compressed sensing (CS) technologies if possible. Third, participant number was relatively limited with various pathological results. We have not discussed the capacities of two DWI ADCs for each tumor. The relationship between ADCs and tumor grades was not discussed either. Nonetheless, our preliminary data demonstrate the feasibility and potential benefits of TGSE-PROPELLER-DWI for orbital tumors in clinical practice. Moreover, further studies with a larger sample size with a broad spectrum of pathologies are still needed.

In conclusion, compared with rs-EPI, TGSE-PROPELLER-DWI showed minimized geometric distortions and susceptibility artifacts and significantly improved the image quality for characterizing orbital tumors. TGSE-PROPELLER-DWI is feasible for depicting orbital tumors and achieves comparable diagnostic performance in differentiating malignant and benign orbital tumors.

## Data Availability Statement

The original contributions presented in the study are included in the article/supplementary material, further inquiries can be directed to the corresponding author/s.

## Ethics Statement

The studies involving human participants were reviewed and approved by the Medical Ethics Committee of Huazhong University of Science and Technology. Written informed consent to participate in this study was provided by the participants’ legal guardian/next of kin. Written informed consent was obtained from the individual(s) for the publication of any potentially identifiable images or data included in this article.

## Author Contributions

QF and FY: conception and design. QF, Y-HG, and KZ: administrative support. QF, X-CK, D-XL, and C-SZ: provision of study materials or patients. QF and X-CK: collection and assembly of data. QF, Z-QL, and FY: data analysis and interpretation. All authors contributed to study design, manuscript writing, study selection, data analysis, study quality evaluating, manuscript revising, and read and approved the final manuscript.

## Conflict of Interest

KZ was employed by the company Siemens Shenzhen Magnetic Resonance Ltd. China. Y-HG was employed by the company Siemens Healthcare Ltd. China. The remaining authors declare that the research was conducted in the absence of any commercial or financial relationships that could be construed as a potential conflict of interest.

## Publisher’s Note

All claims expressed in this article are solely those of the authors and do not necessarily represent those of their affiliated organizations, or those of the publisher, the editors and the reviewers. Any product that may be evaluated in this article, or claim that may be made by its manufacturer, is not guaranteed or endorsed by the publisher.
